# A new species of *Trichoderma hypoxylon* harbours abundant secondary metabolites

**DOI:** 10.1038/srep37369

**Published:** 2016-11-21

**Authors:** Jingzu Sun, Yunfei Pei, Erwei Li, Wei Li, Kevin D. Hyde, Wen-Bing Yin, Xingzhong Liu

**Affiliations:** 1 State Key Laboratory of Mycology, Institute of Microbiology, Chinese Academy of Sciences (CASIM), No. 3 Park 1, West Beichen Road, Chaoyang District, Beijing 100101, China; 2 Center of Excellence in Fungal Research, and School of Science, Mae Fah Luang University, Chiang Rai, 57100, Thailand; 3 Joint Laboratory of Applied Microbial Technology, CASIM and Institute of Biology limited Liability Company, Henan Academy of Sciences, Zheng Zhou, 45002, China

## Abstract

Some species of *Trichoderma* are fungicolous on fungi and have been extensively studied and commercialized as biocontrol agents. Multigene analyses coupled with morphology, resulted in the discovery of *T. hypoxylon* sp. nov., which was isolated from surface of the stroma of *Hypoxylon anthochroum*. The new taxon produces *Trichoderma*- to *Verticillium*-like conidiophores and hyaline conidia. Phylogenetic analyses based on combined ITS, TEF1-α and RPB2 sequence data indicated that *T. hypoxylon* is a well-distinguished species with strong bootstrap support in the polysporum group. Chemical assessment of this species reveals a richness of secondary metabolites with trichothecenes and epipolythiodiketopiperazines as the major compounds. The fungicolous life style of *T. hypoxylon* and the production of abundant metabolites are indicative of the important ecological roles of this species in nature.

Traditionally the taxonomy of species of *Trichoderma* was based on morphology. Most species in this genus are usually fast growing, produce highly branched conidiophores with cylindrical to nearly subglobose phialides and ellipsoidal to globose conidia^1^,^2^,^3^,^4^. However, high morphological homoplasy in sexual state makes identification difficult, and the importance of sequence data have been increased^5^,^6^. Based on the combined phenotypic and phylogenetic analysis, about 260 species have been recognized and accepted^5^,^6^,^7^,^8^,^9^,^10^. The internal transcript spacers (ITS), translation elongation factor 1-alpha (TEF1-α) and largest subunit of RNA polymerase II (RBP2) genes are more available to recognize species within *Trichoderma*^5^,^9^,^10^. Phylogenetic analysis based on a combination of ITS, TEF1-α and RBP2 are recommended solve the problem of *Trichoderma* species complex and reveal taxonomic deversity^2^,^6^,^7^,^8^,^9^,^10^,^11^.

Fungi associated with other fungi as saprobes, commensals or parasites are termed fungicolous fungi^12^. These fungi usually produce rich secondary metabolites, which have been reported as an important resource for bioactive small molecule discovery such as anti-fungi, tumors, nematodes and bacteria^13^,^14^. Some metabolites produced by the species in this genus play very important ecological roles in nature^15^. *Trichoderma* species are most frequently found in vegetable matter, decaying wood and soil, plant rhizosphere, as well as on other fungi^5^,^6^,^16^. Fungicolous *Trichoderma* species comprise diversity and abundant genes associated with secondary metabolites productivity^15^,^16^. These genes are responsible to a number of secondary metabolites with pharmaceutical and biotechnological importance including peptides, peptaibols, poliketides, pyrones, siderophores and nonvolatile terpenes^14^,^15^,^16^,^17^,^18^,^19^,^20^,^21^,^22^. Some of secondary metabolites are responsible for survival and adaption of their habitat^15^,^16^. For example, peptaibols are small peptides of non-ribosomal origin produced by *Trichoderma*^15^ and have the ability to induce systemic resistance in plants against microbial invasion. Harzianum A is a growth-promoting trichothecene produced by *Trichoderma arundinaceum*^17^.

In the search of novel bioactive compounds, we carried out resource and diversity investigation of fungicolous fungi in China and Thailand. Two isolates of *Trichoderma* were obtained from stroma of *Hypoxylon anthochroum*. They are described, illustrated and named as a new species *Trichoderma hypoxylon*. Its phylogenetic positions were also explored, which inferred from sequence analyses of the combined internal transcribed spacer (ITS), partial RNA polymerase II subunit (RPB2) and translation elongation factor 1 alpha (TEF1-α) exon genes. Detailed comparisons were made between the new taxa and their related fungi. Considering *T. hypoxylon* is a new fungicolous species and its special life style, we reasoned that it is valued to study the chemical profiles to get the linkage to the biological roles.

## Results

### Phylogenetic analyses

**Figure 1:**
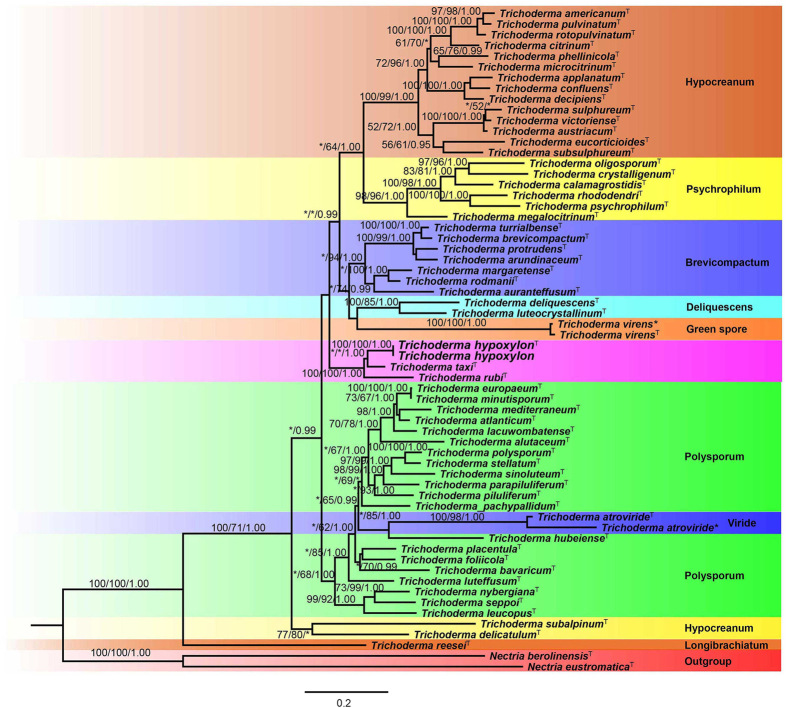
Phylogeny constructed from the combined sequences of ITS, TEF1-α and RBP2. The tree is rooted to *Nectria berolinensis* and *Nectria eustromatica*. MPBP above 50% (left) MLBP above 50% (midle) BIPP above 95% (right) are indicated at the nodes. New species proposed are indicated in boldface.

Alignment results show that the sequences ITS, RPB2 and TEF1-α of *T. hypoxylon* are less than 97% similar to other *Trichoderma* species. Based on phylogenetic analysis of single gene of ITS, RPB2 and TEF1-α, *T. hypoxylon* formed a clade with *Trichoderma taxi* and *Trichoderma rubi*. The position of this clade showed closed relationship with section Hypoceanum, Polysporum, Psychrophila and other species in *Trichoderma* (Fig. S1, and Fig. S2). The phylogenetic analysis of RPB2 showed that *T. hypoxylon* grouped with the Polysporum section (Fig. S3). Therefore, the combination matrix included 60 ingroup taxa of *Trichoderma* which are phylogenetically close to *T. hypoxylon*. This data matrix comprised 2839 characters. Bayesian inference (BI), maximum likelihood (ML) and maximum parsimony (MP) trees generated shared the same topology. In MP analyses, 1642 (57.8%) characters are constant, 216 (7.6%) characters are parsimony-uninformative, and 981 (34.6%) characters are parsimony informative. In MP analyses, 1642 characters were constant, 981 were parsimony-informative, and 216 variable characters were parsimony-uninformative. Maximum likelihood tree was Presented (Fig. 1).

Based on the analyses, 60 strains of *Trichoderma*, including our new species *T. hypoxylon*, formed a strongly supported group (MPBP/MLBP/BIPP = 100%/100%/1.00). They also clustered in 7 recognized subclades and a new subclade including *T. hypoxylon*, Brevicompactum (MLBP/BIPP = 94%/1.00), Deliquescens (MPBP/BIPP = 100%/1.00), Hypoceanum (MPBP/MLBP/BIPP = 100%/99%/1.00), Polysporum (BIPP = 1.00), Psychrophila (MPBP/MLBP/BIPP = 98%/96%/1.00), and green spore sections (MPBP/MLBP/BIPP = 100%/100%/1.00), whereas two strain of *Trichoderma atroviride* Bissett positioned inside Polysporum section. The tree topology is basically congruent with previous reports^6^.

The new species *T. hypoxylon* (CGMCC 3.17906, CGMCC 3.17907), and *T. taxi* and *T. rubi* formed a clade independent from other *Trichoderma* species with strong support (MPBP/MLBP/BIPP = 100%/100%/1.00). However, *T. hypoxylon* isolates were distinguished from *T. axi* and *T. rubi* with high support (MPBP/MLBP/BIPP = 100%/100%/1.00).

### Taxonomy

***Trichoderma hypoxylon*** Jing Z. Sun, Xing Z. Liu & K.D. Hyde, ***sp. nov.***

*Index Fungorum number*: IF552046, *Facesoffungi number*: FoF: 02075, Figs 2, 3, 4.

**Type:—**HMAS 246918

Colonizing or hyperparasitic on stroma of *Hypoxylon anthochroum.* Sexual morph Undetermined. Asexual morph forming *Acremonium*- to *Verticillium*-like conidiophores and hyaline conidia. Phialides lageniform to cylindrical, hyaline, 4.5–12 × 3–3.5 μm (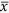 = 6.5 ± 1.4 × 3.2 ± 0.25 μm, n = 30); l/w 2.5–4(−4.5) (n = 30). Mature conidia forming a head at the apex of conidiophores, hyaline, smooth-walled, unicellular, oboviod and mostly 2.8–4.3 × 2.1–2.4 μm (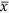 = 3.8 ± 0.4 × 2.2 ± 0.10 μm, n = 30), l/w 1–2(−2.2) (n = 30). Slow growing on PDA, SNA and CMD; PDA colonies especially dense, whitish; SNA and CMA colonies consisting of concentric rings with irregular outline when cultured in darkness at 30 °C.

**Figure 4:**
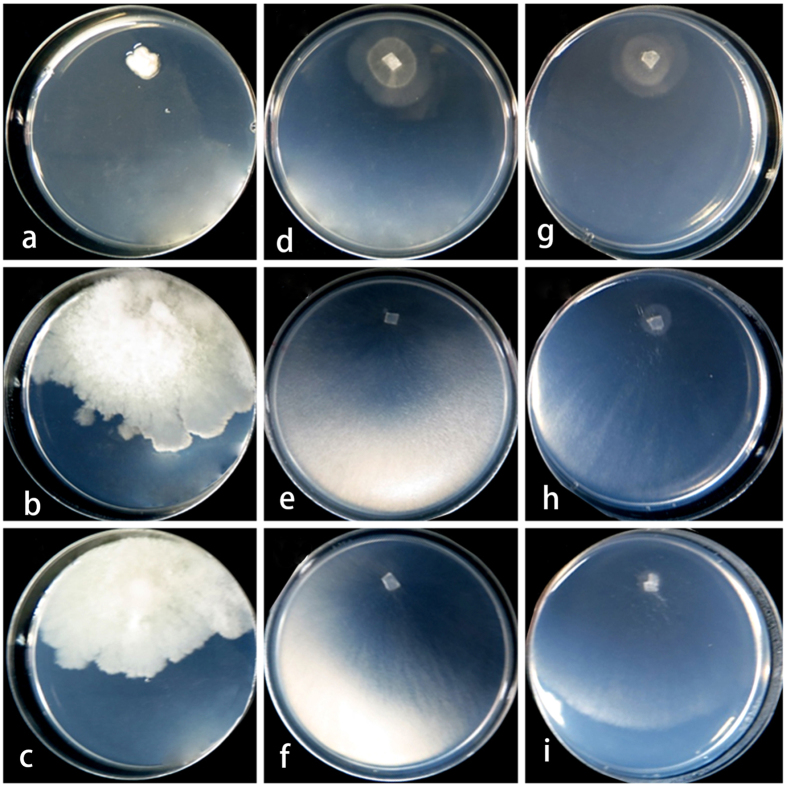
Cultures of *Trichoderma hypoxylon* on PDA, CMD and SNA at different temperature after 10 days. (**a**–**c**) Colony on PDA at 30 °C, 25 °C and 20 °C seperately; (**d**–**f**) colony on CMD at 30 °C, 25 °C and 20 °C seperately; (**g**–**i**) colony on SNA at 30 °C, 25 °C and 20 °C seperately.

Colonies on PDA after 10 d at 20, 25 and 30 °C dense (Fig. 4a–c), with a thick white layer of cotton-like aerial mycelia, forming concentric rings at 20 and 25 °C, however not forming concentric rings at 30 °C, odour woody, and agar not pigmented. Conidia formed within 10 d in the aerial mycelium, mature conidia gathered at the apex of conidiophores, hyaline, smooth walled, 1-celled, oboviod and mostly 2.8–4.3 × 2.1–2.4 μm (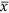 = 3.8 ± 0.4 × 2.2 ± 0.10 μm, n = 30), l/w 1.2–2.2 (−2.5) (n = 30).

Colony on CMD after 10 d at 20, 25 and 30 °C flat (Fig. 4d–f), with a thin white layer of mycelia, not forming concentric rings and conidia at 20 and 25 °C, however forming concentric rings at 30 °C, no distinctive odour, agar not pigmented. Conidiophores *Acremonium*- to irregularly *Verticillium*-like; phialides lageniform to cylindrical, hyaline, 8.5–19.5 × 2–2.51 μm (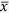 = 13.5 ± 1.4 × 2.1 ± 0.18 μm n = 30), l/w 3.5–9(−9.5) (n = 30); Conidia formed after 20 d, mature conidia gathered at the apex of conidiophores hyaline, smooth walled, one-celled, oval, 4.8–6.3 × 3.0–3.4 μm (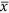 = 4.9 ± 0.4 × 3.1 ± 0.1 μm, n = 30), l/w 1.2–1.6 (−2.0) (n = 30).

Colony on SNA after 10 d at 20, 25 and 30 °C flat (Fig. 4g–i), with a thin white layer of mycelia, not forming concentric rings and conidia at 20 and 25 °C, however forming concentric rings at 30 °C, no distinctive odor, agar not pigmented.

Colony radius on PDA after 72 h at 20 °C, 28.2–30.9 mm; 25 °C, 33.4–36.3 mm; 30 °C, 0.32–1 mm; and 35 °C, 0 mm (n = 5). Colony radius on CMD after 72 h at 20 °C. 41.2–46.1 mm; 25 °C. 37.4–38.3 mm; 30 °C, 8.32–9.12 mm; and 35 °C 0 mm (n = 5). Colony radius on SNA after 72 h at 20 °C, 24.9–26.8 mm; 25 °C, 27.1–29.2 mm; 30 °C, 10.4–12.1 mm, and 35 °C, 0 mm (n = 5).

**Etymology:—***hypoxylon* refers to the genus of host fungus *Hypoxylon anthochroum*.

**Distribution:**—Chiang Rai, Thailand

**Host:**—*Hypoxylon anthochroum*, a saprobic fungus on dead wood.

**Material examination:** Thailand, Chiang Rai Province, isolated from the stroma of *Hypoxylon anthochroum*, 5 May, 2014. MFLU16–1263 (Holotype, dried culture MFLU 16–1263!), HMAS 246918 (Isotype, dried culture HMAS 246918!) ex-type living culture, MFUCC 15–0683, CGMCC 3.17906, CGMCC 3.17907.

*Notes*: The asexual morph of *Trichoderma hypoxylon* produces *Acremonium*- to *Verticillium*-like conidiophores and hyaline conidia. Phylogenetically *T. hypoxylon* is related to *T. taxi* and *T. rubi* and together formed a new independent clade distinguished other *Trichoderma* species (Fig. 1). However, *T. hypoxylon* can be phylogenetically distinguished from these species. The conidia of *T. hypoxylon* (2.8–4.3 × 2.1–2.4 μm) are also longer than *T. taxi* (2.4–3.1 × 2.0–2.5 μm)^23^ and *T. rubi* (2.3–3.3 × 2.0–2.7 μm)^6^. Meanwhile, *T. hypoxylon* did not produce pigment on medium, whereas *T. rubi* produced brownish pigment and yellow crystals^6^.

### Secondary metabolite analysis and characterization of compounds

**Figure 5:**
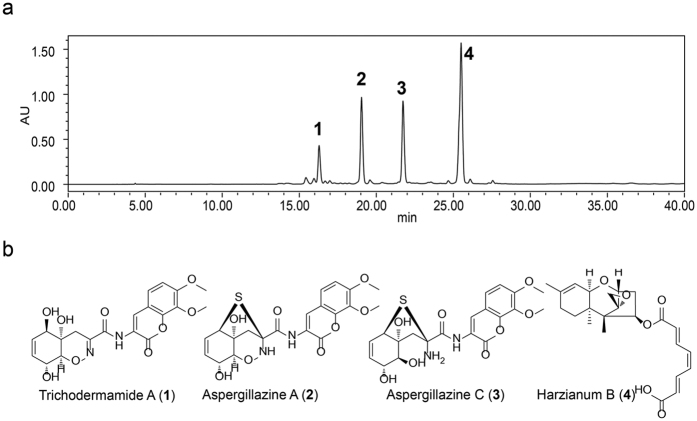
Secondary metabolite assessment of *Trichoderma hypoxylon*. (**a**) HPLC analysis of secondary metabolite production under 330 nm wavelength. The strain was grown for 14 days at 25 °C on PDA media. (**b**) Four characterized compounds in this study. 1: trichodermamide A, 2: aspergillazine A, 3: aspergillazine C, 4: harzianum B.

HPLC analyses of extracts of *T. hypoxylon* cultivated on PDA medium were carried out to assess the production of secondary metabolites. Four major peaks with high yields were obtained by HPLC chromatography (Fig. 5A). To characterize compounds from *T. hypoxylon*, 1 liter fermentation on PDA medium were performed. After the semi-preparative reversed-phase HPLC separation step, we isolated four known compounds trichodermamide A (**1**), aspergillazine A (**2**), aspergillazine C (**3**) and harzianum B (**4**) (Fig. 5B). The assignments of four compounds were based on the published data of proton NMR^24^,^25^,^26^,^27^. Notably, the yields of harzianum B were 50 mg out of 330 mg crude extracts.

## Discussion

Traditionally, delimitation of *Trichoderma* species was mainly based on the morphology^1^, although, it could not well explain the taxonomic position of these species^6^,^9^. Phylogenetic analysis has resulted in the discovery of many new species and has been extensively used in fungal taxonomy^28^. Presently, more than 258 species of *Trichoderma* are accepted based on phylogenetic analysis^5^,^10^. However, many species of *Trichoderma* remain to be discovered and described^5^,^6^. To explore the taxonomic position of *T. hypoxylon*, phylogenetic tree containing all species of *Trichoderma* species was constructed and the putative position of *T. hypoxylon* was shown (Figs S1–S3). The single gene of ITS, RPB2 and TEF1-α could distinguish *T. hypoxylon* from other *Trichoderma* species, the results suggested that these three genes are effective in taxonomy of *Trichoderma*^5^,^6^,^9^. However, the single gene could not well delimit Longibrachiatum, Viride and some other sections in genus of *Trichoderma*. Multigene analysis was a popular and feasible approach to solve the problem^5^,^6^,^10^.

Based on analyses of the combined sequences of ITS, RPB2 and TEF1-α, 56 currently known species in *Trichoderma* clustered together (Fig. 1). Nine subclades, Brevicompactum, Deliquescens, Green spore group, Hypoceanum, Longibrachiatum, Polysporum, Psychrophilum, Viride and a new subclade including *Trichoderma hypoxylon* were recognized, which is basically congruent with the results by Jaklitsch and Voglmayr^6^. The new subclade contained *T. hypoxylon*, *T. taxi* and *T. rubi* together readily distinguished from other *Trichoderma* species^6^. *Trichoderma hypoxylon* clearly differed from *T. taxi* and *T. rubi*, resulting the sequences similarity of RPB2 and TEF1-α are less than 97%.

*Trichoderma rubi* was found as a new saprobe on stems of *Rubus ulmifolius*, *T. taxi* was reported as new endophyte of *Taxus mairei*, whereas *T. hypoxylon* was an inhabitant on stroma of *Hypoxylon anthochroum*. In spite of their ecological niches, it showed closed phylogenetic relationship (Fig. 1, Figs S1–S3) and morphological similarity. However, they are a little different in conidiophores and conidia size, and *T. hypoxylon* did not produce pigment on PDA medium, whereas *T. rubi* produced brownish pigment and yellow crystals on PDA medium^6^.

*Trichoderma* species are a rich source of secondary metabolites^14^,^15^,^16^,^17^,^18^,^19^,^20^,^21^,^22^,^29^, probably resulting their environments adaptation and lifestyles^15^,^16^,^30^. Comparative genome analysis revealed that fungicolous *T. atroviride* and *T. virens* are enriched in secondary metabolism-related genes compared with the biomass-degrading *Trichoderma reesei*^30^. Chemical analysis also showed that these fungicolous species could produce more peptaibols, peptides, polyketides, pyrones, siderophores, terpenoids/steroids than those non-fungicolous *Trichoderma*^15^,^30^. These compounds are ecologically and commercially important for their antimicrobial and anti-cancer properties, as well as their ability to induce systemic resistance in plants against microbial invasion^15^,^17^,^31^. As a new fungicolous species, the chemical diversity of *T. hypoxylon* aroused our attentions. Therefore, the secondary metabolites of this fungus were evaluated. Four major compounds were characterized (Fig. 5). Trichodermamide and aspergillazines are two kind of modified dipeptides^15^,^25^. Trichodermamide A has been found from a marine-derived *T. virens*^24^, it is also be obtained from marine-derived fungi *Spicaria elegans*^25^ and *Neosartorya pseudofischeri*^32^ and endophytic fungus *Trichoderma spirale*^33^. The yield of trichodermamide A produced by these fungi were 1.58 mg/L^24^, 0.73 mg/L^25^, 0.12 mg/L^32^ and 8.98 mg/L^33^ individually. In this study, we found that trichodermamide A was one of major compounds in the terrestrial fungus *T. hypoxylon.* It suggested that *Trichoderma* species are important resource for exploration of trichodermamide. Aspergillazines were firstly reported from a soil fungi *Aspergillus unilaterali*s (MST-F867)^26^, it showed antibacterial and anti-cancer activity^15^,^19^. Aspergillazines A been found from two marine-derived fungi *Spicaria elegans*^26^ and *T. virides*^25^, in which the productive rate of aspergillazines A was 3.17 mg/L^26^ and 0.47 mg/L^25^. This compound has been found co-occurred with trichodermamide A in *T. virides*^25^. Trichothecenes are a well-studied class of sesquiterpene-based mycotoxins^34^. They are potent cytotoxins to eukaryotic cells which are mainly produced by fungal species of *Fusarium*, *Myrothecium* and *Trichoderma* in order Hypocreales^15^,^34^. Harzianum B is one of trichothecenes, which inhibit eukaryotic cell growth^27^ and have the cytotoxic, antibiotic, and anthelmintic activities^35^. It is found that in a *Hypocrea* sp. (sexual morph of *Trichoderma*) strain F000527 yield of 18.13 mg/L^27^, while our results showed harzianum B was extremely high in *T. hypoxylon* with yield of 50 mg/L. The high amount of trichothecenes in *T. hypoxylon*, indicating that it has antagonistic potential against fungal hosts.

## Materials and Methods

### Isolates and specimens

Samples were collected on 5 May 2014 in Chiang Mai Province, Thailand. *Trichoderma* strains were isolated from the host by single spore isolation as detailed in Chomnunti *et al.*^36^. The holotype is deposited in the Herbarium of Mae Fah Luang University and the isotype in the Herbarum of Mycology, Chinese Academy of Science (HMAS, Beijing, China). Ex-type living cultures are deposited in the Culture Collection of Mae Fah Luang University (MFLUCC, Chiang Rai, Thailand) and the China General Microbiological Culture Collection Center (CGMCC, Beijing, China). Facesoffungi and Index Fungorum numbers are registered as explained in Jayasiri *et al.*^37^ and Index Fungorum^38^.

### Morphological characterization

Methods and morphology were described basically following counterparts by Jaklitsch & Voglmayr^6^ Colony radius and characteristics were determined on PDA as detailed in Manamgoda *et al.*^6^ cornmeal dextrose agar (CMD; Difco cornmeal agar + 2% w/v dextrose)^10^ and a defined low nutrient agar (SNA)^4^ at 20, 25 and 30 °C in darkness for 7 d, then exposed to artificial light to stimulate conidia formation until 10 d. Microscopic observations and measurements were made from preparations mounted in 50% lactic acid. Photographs were taken with a Nikon DS–Fi2 CCD (Nikon, Japan) connected to a Nikon 80i microscope (Nikon, Japan) for anatomical structures. The statistics presented here are based on measurement of 30 mature conidia (±S.D.) and 30 phialides (±S.D.) at 100× magnification. To assess and describe their structure and morphology of conidiophores were taken from the edge of conidiogenous pustules or fascicles. Conidia were studied from cultures after 10 d of incubation.

### DNA extraction, PCR amplification and sequencing

Approximately 50 mg of fungal material from each culture was placed in 600 μl of 2% CTAB buffer and ground with a plastic pestle. Genomic DNA was extracted using a modified CTAB extraction protocol^39^. Three primer pairs, ITS5 and ITS4^40^, fRPB2–5 f and fRPB2–7cr^23^, EF983F and EF2218R^41^ were separately used to amplify fragments of ITS, RPB2 and TEF1-α. Each PCR was performed on a Votix thermal cycler (Bio-Rad, CA, USA) using easy tag (Tiangen, Beijing, China) in a final volume of 50 μl containing 10 μmol of each primer and 2 μl of DNA (10 ng/L). Reactions were run with positive and negative controls to ensure accuracy and to detect contamination. Automated sequencing was performed by Sino Geno Max Co. (Beijing, China). The sequences used in this study are deposited in GenBank under the accession number provided in Table S1.

### Phylogenetic analysis

The ITS and TEF1-α data sets used sequences of isolates CGMCC 3.17906, CGMCC 3.17907 and reference sequences were downloaded from GenBank (Table S1). The ITS, TEF1-α and RPB2 data sets were aligned by MAFFT ver.7.03 using the Q-INS-I strategy, individually^42^. The ambiguous areas of alignment were located and removed using Gblocks 0.91b^43^. Previous phylogenetic analysis of ITS, RPB2 and TEF1-α sequence data from 260 *Trichoderma* species was conducted seperately with *N. berolinensis* and *N. eustromatica* as outgroup. Then, single and combined genes analyses of ITS, TEF1-α and RPB2 sequence data of 60 phylogenetic closed *Trichoderma* species in Brevicompactum, Deliquescens, Hypoceanum, Longibrachiatum, Polysporum, Psychrophila and green spore sections were carried out. *N. berolinensis* and *N. eustromatica* were arranged as outgroup taxa.

Maximum parsimony (MP) analysis was conducted by PAUP 4.0b10^44^ using a heuristic search with tree-bisection-reconnection branch swapping. All characters were treated as unordered and unweighted, gaps were treated as missing data, sequences were auto-increased and Maxtrees was 5,000. Topological confidence of resulted trees was tested by bootstrap proportion with 1,000 replicates, each with 100 replicates of random addition. Bootstrap proportion (BP) higher than 50% from maximum parsimony analysis from PAUP are given.

Maximum-likelihood (ML) analysis was performed in RAxML^45^ implemented in raxml GUI v.1.3^46^. GTRGAMMAI was specified as the model. The analysis was run with a rapid bootstrap analysis using a random start with rapid bootstrap analysis with 1,000 replicates. Bootstrap proportion (BP) higher than 50% from maximum likelihood analysis from RAxML are given.

Bayesian Inference (BI) analysis was performed with MrBayes 3.1.2^47^ using Markov chain Monte Carlo (MCMC) algorithm. Appropriate nucleotide substitution models was determined by MrModeltest 2.3^48^ and the best fit model “GTR + I + G” was selected by Akaike Information Criterion for the investigated data set. Six chains (one cold and three heated) of 135,000 Markov chain Monte Carlo generations were run, sampling every 100 generation resulting in 1,350 total trees (in two simultaneous analyses). The initial 337 trees (25%) were discarded as burn-in phase of the analyses, and the remaining trees in each analysis were used to calculate posterior probabilities (PP) in the majority rule consensus tree^49^, posterior probabilities greater than 0.95 are given.

All trees were viewed in TreeView 1.6.6^50^ and revised in Adobe Illustrator CS5.

### Analytical methods and equipment overview

^1^H-NMR spectra were recorded on a Bruker Avance-500 spectrometer using TMS as internal standard, and chemical shifts were recorded as *δ* values. ESI-MS utilized on an Agilent Accurate-Mass-QTOF LC/MS 6520 instrument. HPLC analysis was performed on a Waters HPLC system (Waters e2695, Waters 2998, Photodiode Array Detector) using an ODS column (C18, 250 × 4.6 mm, YMC Pak, 5 μm) with a flow rate of 1 mL/min.

### Fermentation and isolation

The fungal strain was cultured on 20 slants of potato dextrose agar at 25 °C for seven days. The fermented PDA substrate was extracted repeatedly with ethyl acetate by exhaustive maceration (4 × 200 mL), and the organic solvent was evaporated to dryness under vacuum to afford the crude extract (330 mg). The residue was fractionated by Sephadex LH-20 CC using CH_2_Cl_2_: Acetone (V:V = 1:1) elution to obtain fifteen fractions 1–15. The fractions 3–7 (200 mg) was separated by semi-preparative RP-HPLC (Waters Symmetry PrepTM C18 column; 7 μm; 7.8 × 300 mm; 45% MeOH in H_2_O over 45 min; 2 mL/min) to afford 4 (*t*_R_ 34.5 min; 50.0 mg). The fraction 11 (35 mg) was separated by semi-preparative RP-HPLC (Waters Symmetry PrepTM C18 column; 7 μm; 7.8 × 300 mm; from 22% CH_3_CN to 30% CH_3_CN in H_2_O over 45 min; 2 ml/min) to afford **1** (*t*_R_ 18.8 min; 5.0 mg), **2** (*t*_R_ 22.4 min; 8.0 mg), and **3** (*t*_R_ 41.1 min; 4.5 mg).

#### Trichodermamide A (1)

White powder (MeOH)^25^,^26^; ^1^H NMR (500 MHz, in Acetone-*d*_6_) δ_H_: 2.72 (*dd*, *J* = 19.4, 2.2, H-3a); 2.28 (*d*, *J* = 19.4, H-3b); 4.48 (*m*, H-5); 5.60 (*ddd*, *J* = 10.4, 2.0, 2.0, H-6); 5.55 (*ddd*, *J* = 10.4, 2.0, 2.0, H-7); 4.24 (*m*, H-8); 4.15 (*dd*, *J* = 7.8, 2.2, H-9); 8.58 (*s*, H-3′); 7.41 (*d*, *J* = 8.8, H-5′); 7.12 (*d*, *J* = 8.8, H-6′); 3.96 (*s*, 7′-OCH_3_); 3.91 (*s*, 8′-OCH_3_); 9.40 (*s*, CONH); Positive ESIMS: *m/z* 433.1 [M+H]^+^.

#### Aspergillazine A (2)

Yellow powder (MeOH)^26^,^27^; ^1^H NMR (500 MHz, in Acetone-*d*_6_) δ_H_: 3.14 (*d*, *J* = 11.7, H-3a); 2.42 (*d*, *J* = 11.7, H-3b); 4.16 (d, *J* = 4.9, H-5); 5.95 (*dd*, *J* = 10.0, 4.9, H-6); 6.07 (*dd*, *J* = 10.0, 4.9, H-7); 4.33 (*m*, H-8); 4.23 (br *s*, H-9); 6.90 (*s*, H-3′); 7.09 (*d*, *J* = 8.8, H-5′); 6.69 (*d*, *J* = 8.8, H-6′); 3.90 (*s*, 7′-OCH_3_); 3.83 (*s*, 8′-OCH_3_); 9.38 (*s*, CONH); 9.90 (*s*, -O-NH); Positive ESIMS: *m/z* 449.1 [M + H]^+^.

#### Aspergillazine C (3)

Yellow powder (MeOH)^27^; ^1^H NMR (500 MHz, in Acetone-*d*_6_) δ_H_: 2.96 (*d*, *J* = 13.9, H-3a); 2.40 (*d*, *J* = 13.9, H-3b); 4.31 (*m*, H-5); 5.42 (*dd*, *J* = 10.0, 1.8, H-6); 5.49 (*dd*, *J* = 10.0, 1.9, H-7); 4.16 (*dd*, *J* = 7.8, 1.2, H-8); 3.77 (*d*, *J* = 8.0, H-9); 6.85 (*s*, H-3′); 7.11 (*d*, *J* = 8.8, H-5′); 6.69 (*d*, *J* = 8.8, H-6′); 3.90 (*s*, 7′-OCH_3_); 3.84 (*s*, 8′-OCH_3_); Positive ESIMS: *m/z* 451.1 [M + H]^+^.

#### Harzianum B (4)

White powder (MeOH)^28^; ^1^H NMR (500 MHz, in CDCl_3_) δ_H_: 3.84 (*d*, *J* = 5.2, H-2); 2.57 (*dd*, *J* = 15.5, 7.8, H-3a); 2.03 (*ddd*, *J* = 15.5, 5.3, 3.6, H-3b); 5.65 (*dd*, *J* = 7.8, 3.6, H-4); 1.92‒1.96 (*m*, H-7a); 1.40‒1.43 (*m*, H-7b); 1.97‒1.99 (*m*, H-8); 5.41 (*d*, *J* = 5.8, H-10); 3.63 (*d*, *J* = 5.8, H-11); 3.13 (*d*, *J* = 4.0, H-13a); 2.83 (*d*, *J* = 4.0, H-13b); 0.72 (*s*, H_3_-14); 0.95 (*s*, H_3_-15); 1.71 (*s*, H_3_-16); 6.03 (*d*, *J* = 15.4, H-2′); 7.32 (*dd*, *J* = 15.4, 10.2, H-3′); 6.61 (*dd*, *J* = 10.2, 10.2, H-4′); 6.64 (*dd*, *J* = 10.2, 10.2, H-5′); 7.32 (*dd*, *J* = 15.4, 10.2, H-6′); 6.07 (*d*, *J* = 15.4, H-7′); Positive ESIMS: *m/z* 401.2 [M + H]^+^.

## Additional Information

**How to cite this article**: Sun, J. *et al.* A new species of *Trichoderma hypoxylon* harbours abundant secondary metabolites. *Sci. Rep.* **6**, 37369; doi: 10.1038/srep37369 (2016).

**Publisher’s note:** Springer Nature remains neutral with regard to jurisdictional claims in published maps and institutional affiliations.

## Supplementary Material

Supplementary Information

## Figures and Tables

**Figure 2:**
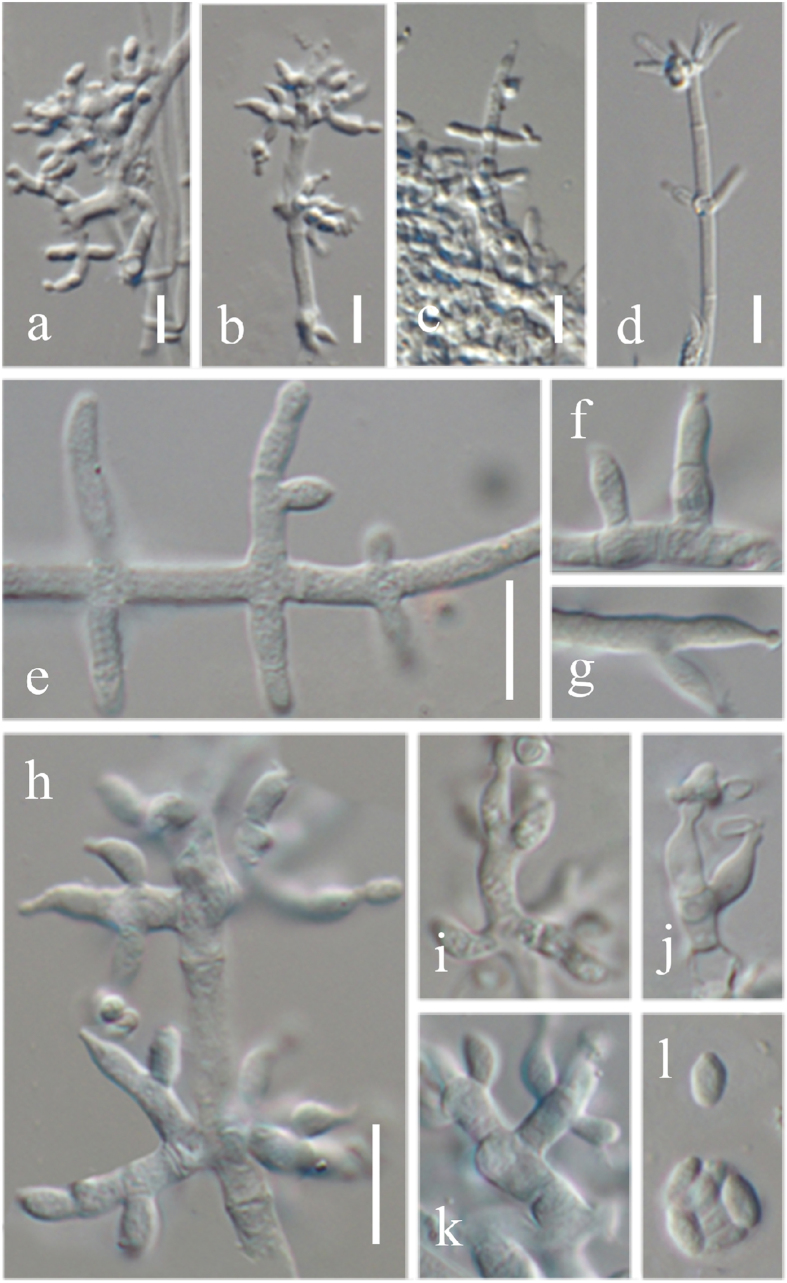
Morphologicial characteristics of *Trichoderma hypoxylon* (ex-type CGMCC 3.17906) on PDA. (**a**–**c**) and (**h**–**k**) conidiophores with condia; (**d**–**g**) conidiophores without condia; l, conidia; bar = 10 μm.

**Figure 3:**
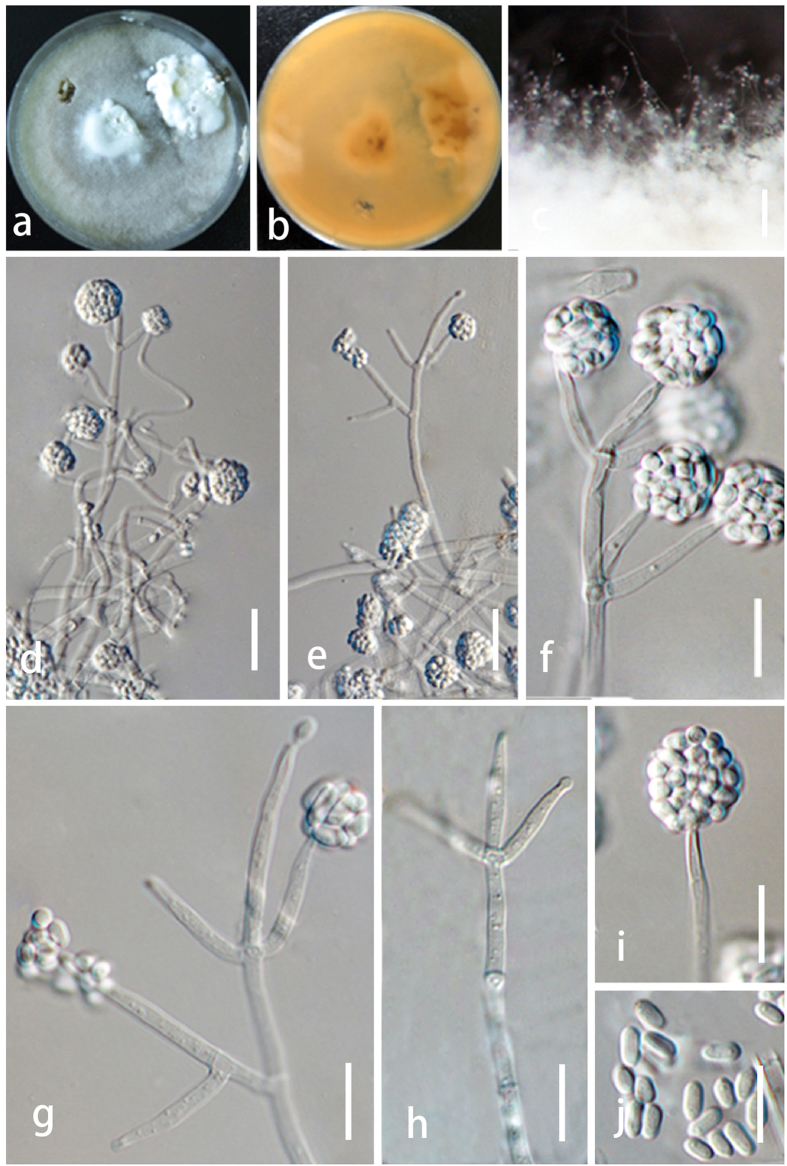
Morphologicial characters of *Trichoderma hypoxylon* (ex-type CGMCC 3.17906) on CMD after 30 days. (**a**) Forward of the colony; (**b**) reverse of the colony; (**c**) mycelia; (**d**,**e**,**i**) conidiophores with condia; (**h**) conidiophores without condia; (**j**) conidia; c = 1000 μm; (**d**) e = 20 μm; c f–j = 10 μm.
