# Supplementary material for: A new species of Trichoderma hypoxylon harbours abundant secondary metabolites
Source: Sci Rep. 2016 Nov 21;6:37369. doi: 10.1038/srep37369 (PMC5116760; doi:10.1038/srep37369)

A new species of *Trichoderma hypoxylon* harbours abundant secondary metabolites

Jingzu Sun[1, 2], Yunfei Pei[1†], Erwei Li[1], Wei Li[1], Kevin D. Hyde[2], Wen-Bing Yin[1*] & Xingzhong Liu[1, 3*]

[1]State Key Laboratory of Mycology, Institute of Microbiology, Chinese Academy of Sciences (CASIM), No. 3 Park 1, West Beichen Road, Chaoyang District, Beijing 100101, China

[2]Center of Excellence in Fungal Research, and School of Science, Mae Fah Luang University, Chiang Rai, 57100, Thailand

[3]Joint Laboratory of Applied Microbial Technology, CASIM and Institute of Biology limited Liability Company, Henan Academy of Sciences, Zheng Zhou, 45002, China

†Current address: National Institutes for Food and Drug Control, No. 2, Tiantan Xili, Dongcheng District, Beijing 100050, China

* Corresponding author

Telephone: +86 10 6480 7512 Fax: +86 10 6480 7505

E-mail address: liuxz@im.ac.cn (X.Z. Liu); yinwb@im.ac.cn (W.B. Yin)

Supplementary Figure Legends

Supplementary Figure S1. Phylogeny constructed based on ITS sequence. The tree is rooted to *Nectria berolinensis* and *Nectria eustromatica*. Maximum likelihood bootstrap propotion higher than 50% are given.

Supplementary Figure S2 Phylogene constructed based on TEF1-α sequence. The tree is rooted to *Nectria berolinensis* and *Nectria eustromatica*. Maximum likelihood bootstrap propotion higher than 50% are given.New species proposed is indicated in boldface.

Supplementary Figure S3 Phylogene constructed based on RPB2 sequence. The tree is rooted to *Nectria berolinensis* and *Nectria eustromatica*. Maximum likelihood bootstrap propotion higher than 50% are given.New species proposed is indicated in boldface.

Supplementary Table S1 Species and sequences used in phylogenetic analyses

Species	Strain	Genbank accession No.		
		ITS	TEF1-a	RPB2
Trichoderma alutaceum	CBS 120535^T	FJ860725	FJ179567	FJ179600
Trichoderma americanum	G.J.S. 92-93^T	DQ835410	DQ835489	DQ835455
Trichoderma applanatum	HMAS 252871^T	KJ783286	KJ634754	KJ634721
Trichoderma arundinaceum	ATCC 90237^T	EU330927	EU338291	EU338326
Trichoderma atlanticum	C.P.K. 1896^T	FJ860780	FJ860648	FJ860545
Trichoderma atroviride	IMI 206040*	AF278795	XM_014085582	XM_014087137
Trichoderma atroviride	CBS 142.95^T	AY380906	AY376051	EU341801
Trichoderma aurantioeffusum	CBS 119284^T	FJ860728	FJ860613	FJ860520
Trichoderma austriacum	CBS 122494^T	FJ860735	FJ860619	FJ860525
Trichoderma bavaricum	CBS 120538^T	FJ860737	FJ860621	FJ860527
Trichoderma brevicompactum	GJS 04-381^T	EU330941	EU338299	EU338317
Trichoderma calamagrostidis	CBS121133^T	FJ860739	FJ860622	FJ860528
Trichoderma citrinum	C.P.K.960^T	NA	FJ860631	FJ179603
Trichoderma confluens	HMAS 244993^T	NA	KT001959	KT001964
Trichoderma crystalligenum	CBS 118980^T	DQ344490	DQ345342	DQ345347
Trichoderma decipiens	G.J.S. 91-101^T	DQ835507	DQ835476	DQ835520
Trichoderma delicatulum	CBS 120631^T	FJ860751	FJ860636	FJ860535
Trichoderma deliquescens	CBS 121132^T	FJ860772	FJ860644	FJ179609
Trichoderma eucorticioides	G.J.S. 99-61^T	DQ835467	DQ835474	DQ835518
Trichoderma europaeum	Hypo 183^T	NA	KJ665474	KJ665261
Trichoderma foliicola	Hypo 645^T	JQ685871	JQ685862	JQ685876
Trichoderma hubeiense	HMAS 252888^T	NA	KT001956	KT001961
Trichoderma hopoxylon	**CGMCC 3.17906^T**	**KU974004**	**KU974001**	**KX500395**
Trichoderma hopoxylon	**CGMCC 3.17907**	**KU974002**	**KU974003**	**KX500396**
Trichoderma lacuwombatense	G.J.S. 99-198^T	DQ083017	KJ665547	KJ665286
Trichoderma leucopus	CBS 122499^T	FJ860764	FJ179571	FJ179605
Trichoderma luteffusum	CBS 120537^T	FJ860773	FJ860645	FJ860543
Trichoderma luteocrystallinum	CBS 123828^T	FJ860774	FJ860646	FJ860544
Trichoderma margaretense	C.P.K. 3127^T	FJ860741	FJ860625	FJ860529
Trichoderma mediterraneum	S347^T		KJ665582	KJ665301
Trichoderma megalocitrinum	B.E.O. 00-09^T	DQ835511	AY225855	AF545563
Trichoderma microcitrinum	G.J.S. 97-248^T	DQ835424	DQ835479	DQ835462
Trichoderma minutisporum	CBS 121276^T	NA	FJ179574	FJ179610
Trichoderma nybergiana	CBS 122496^T	FJ860792	FJ179576	FJ179612
Trichoderma oligosporum	HMAS 252870^T	KJ783296	KJ634764	KJ634731
Trichoderma pachypallidum	CBS 122126^T	FJ860798	FJ860662	FJ860560
Trichoderma parapiluliferum	CBS 120921^T	FJ860799	FJ179578	FJ179614
Trichoderma phellinicola	CBS 119283^T	FJ860808	FJ860672	FJ860569
Trichoderma piluliferum	C.P.K.3143^T	FJ860811	FJ179579	FJ860571
Trichoderma placentula	CBS 120924^T	FJ860813	FJ179580	FJ179616

Species	Strain	Genbank accession No.		
		**ITS**	**TEF1-a**	**RPB2**
Trichoderma polysporum	HMAS 266664^T	KJ783303	KJ634771	KJ634738
Trichoderma protopulvinatum	CBS 739.83^T	FJ860816	FJ860679	DQ835463
Trichoderma protrudens	DIS 119F^T	EU330946	EU338289	EU338322
Trichoderma psychrophilum	C.P.K. 1602	FJ860818	FJ860680	FJ860575
Trichoderma pulvinatum	CBS 121279^T	FJ860820	FJ860683	FJ860577
Trichoderma rhododendri	CBS 119288^T	FJ860822	FJ860685	FJ860578
Trichoderma reesei	QM 6a^T		AF401004	HM182969
Trichoderma rodmanii	CBS 121553^T	FJ860824	FJ860687	FJ860580
Trichoderma rubi	S146^T		KJ665704	KJ665336
Trichoderma seppoi	CBS 122497^T	FJ860834	FJ179582	FJ179618
Trichoderma sinoluteum	HMAS 252868^T	KJ783309	KJ634777	KJ634744
Trichoderma stellatum	G.J.S. 99-222^T	NA	KJ665741	KJ665349
Trichoderma subalpinum	C.P.K. 3126^T	FJ860851	FJ860706	FJ860596
Trichoderma subsulphureum	M141^T	DQ835509	DQ835492	DQ835522
Trichoderma sulphureum	G.J.S. 00-172^T	DQ835510	DQ835493	DQ835523
Trichoderma taxi	ZJUF0986^T	DQ470074	DQ859029	DQ859032
Trichoderma turrialbense	CBS 112445^T	EU330945	EU338284	EU338321
Trichoderma victoriense	G.J.S. 99-130^T	DQ835504	EU338331	EU338336
Trichoderma virens	Gv29-8*		XM_014101441	XM_014102714
Trichoderma virens	DAOM 167652^T	EU330955	AF534619	AF545547
Nectria berolinensis	CBS 127382^T	HM534893	HM534872	HM534883
Nectria eustromatica	CBS 121896^T	HM534896	HM534875	HM534886

Notes: T denotes: Type isolates; * denotes: Genome sequenced isolates; CBS= Centraalbureauvoor Schimmelcultures, Netherlands; CGMCC = China General Microbiological Culture Collection Center, China; Herbarum of Mycology, Chinese Academy of Science (HMAS, Beijing, China)

Supplemental Figure S1

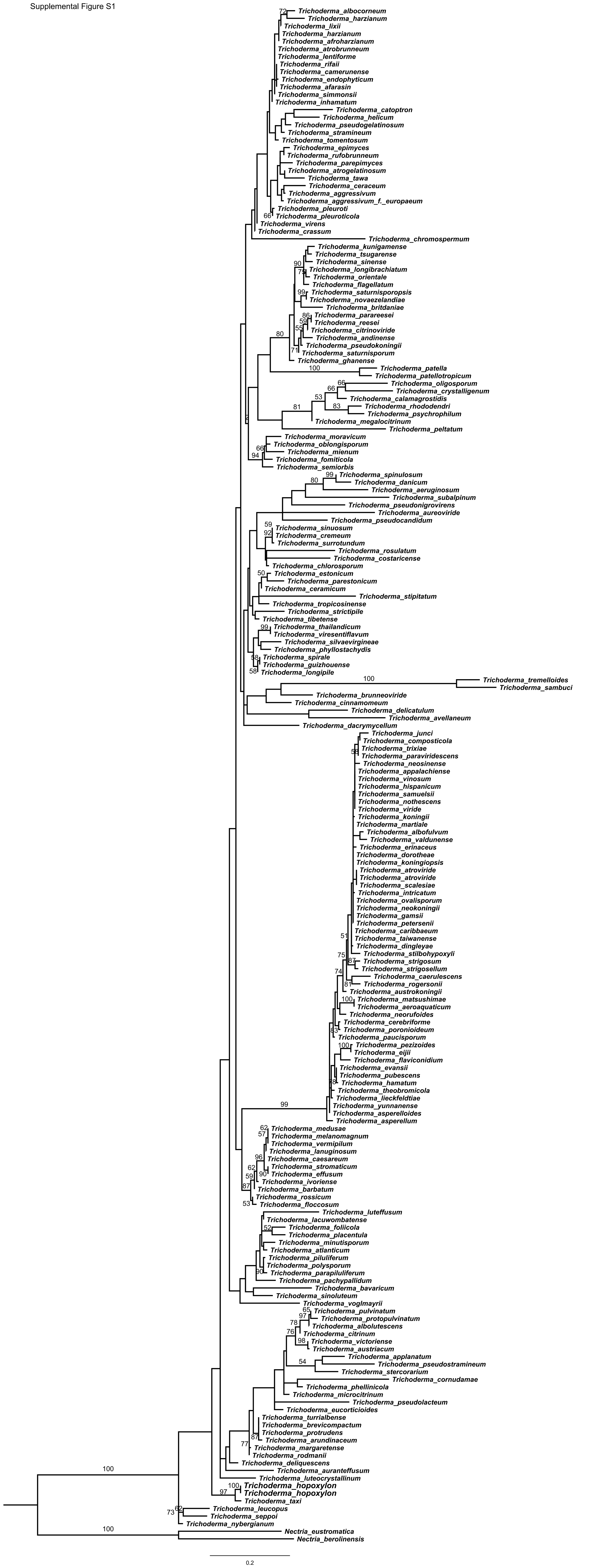

Supplemental Figure S2

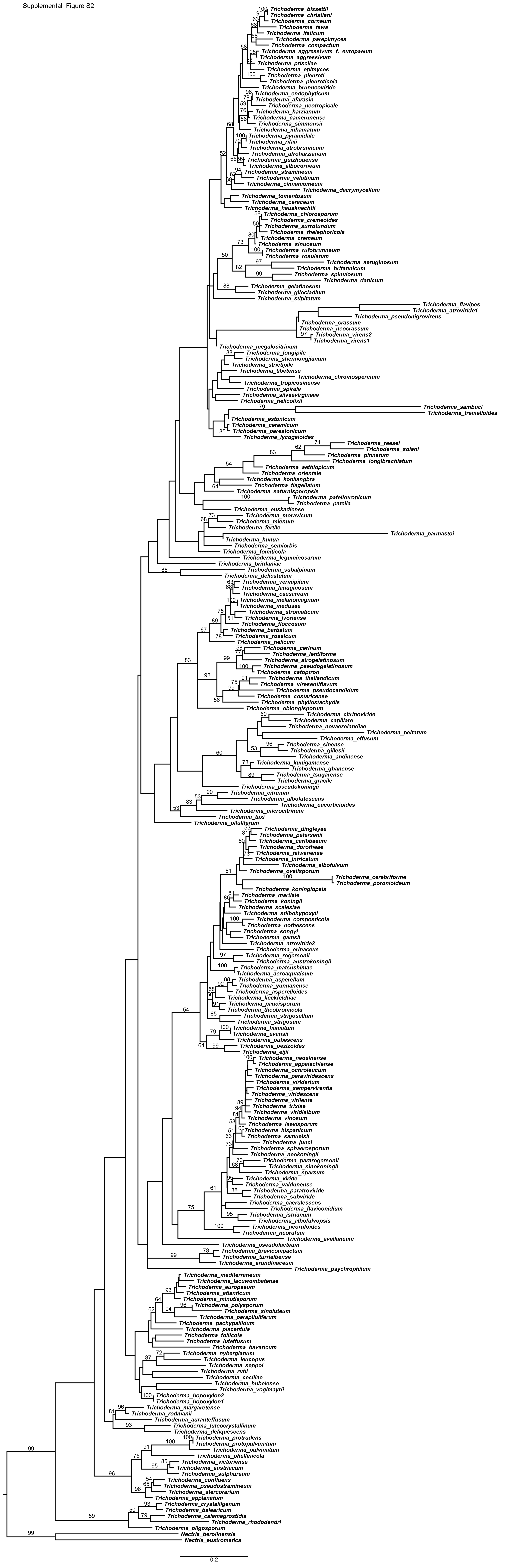

Supplemental Figure S3

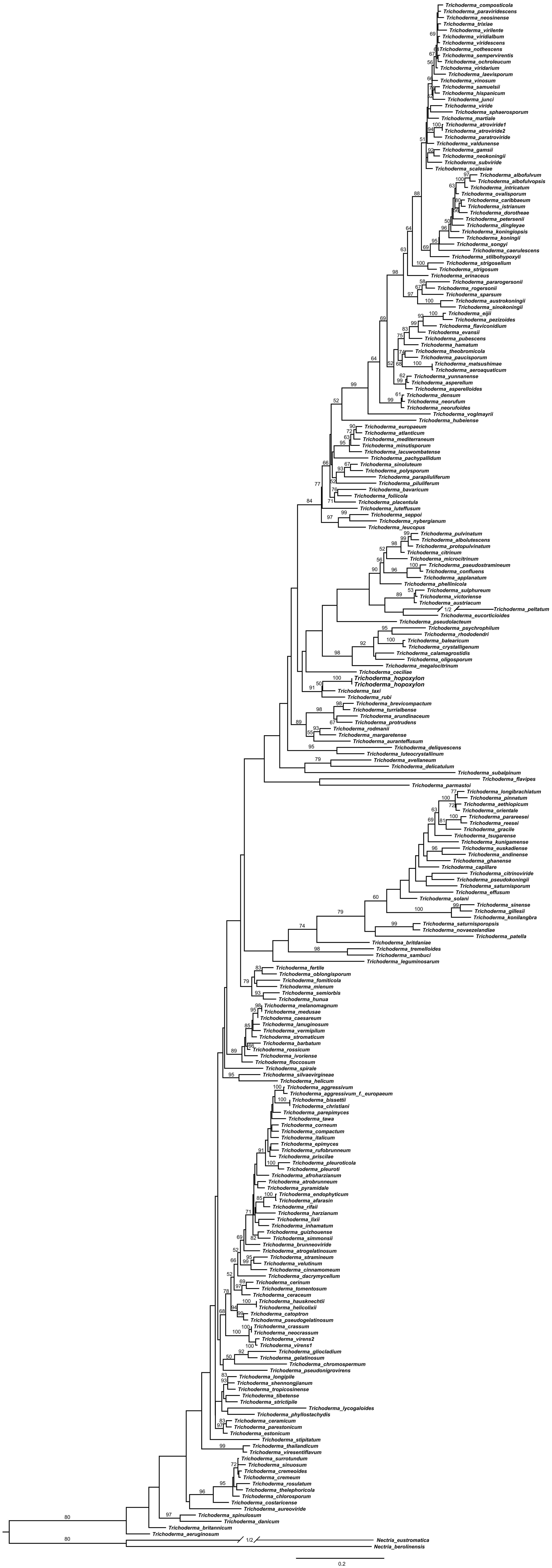

Supplement: Supplementary Information [file srep37369-s1.pdf]
